# A novel technique to improve postoperative stability of orthognathic surgical anterior open bite correction using temporary anchorage devices: a case report

**DOI:** 10.1186/s40902-025-00474-9

**Published:** 2025-08-29

**Authors:** Farhad B. Naini, Nausheen Siddiqui, Aoibhean Wall, Umberto Garagiola, Ashraf Messiha

**Affiliations:** 1 Department of Orthodontics, Maxillofacial Unit, Kingston Hospital, London, United Kingdom; 2 Maxillo-facial and Odontostomatology Unit, University of Milan, Milan, Italy

**Keywords:** Orthognathic surgery, Anterior open bite, Stability, Temporary anchorage devices

## Abstract

**Background:**

Postoperative stability is a significant problem in the orthognathic management of anterior open bite malocclusion. The general tendency of modern preadjusted fixed appliances is towards unwanted and unplanned extrusion of the maxillary incisor and canine teeth as the dental arch is levelling. Following surgical repositioning of the jaws, the relapse potential of the extruded anterior dentition will be to intrude, leading to some reopening of the surgically corrected anterior open bite.

**Case presentation:**

A 19-year-old male white Caucasian patient presented with a clinically significant anterior open bite of predominantly skeletal aetiology. The objective of preoperative levelling in the maxillary dental arch was to avoid any extrusion of the anterior dentition. To achieve this aim, two temporary anchorage devices (TADs) were placed in the maxillary alveolar bone, and relatively passive elastic force was applied from the archwire to the TADs in order to prevent maxillary incisor extrusion during arch levelling. This elastomeric chain was maintained throughout the alignment and levelling of the maxillary dental arch.

The patient had a Le Fort I osteotomy of the maxilla with differential posterior impaction and advancement, and mandibular forward autorotation and small setback of the mandibular body with bilateral sagittal split osteotomy, to achieve a Class I incisor and skeletal position. No vertical movement of the teeth was carried out or required following surgery. The patient was debonded 3 months following surgery and fitted with removable retainers.

Cephalometric superimpositions demonstrated that no extrusion of the anterior maxillary dentition occurred, which is the main parameter to improve postoperative stability of the anterior open bite correction.

**Conclusions:**

To improve the potential stability of anterior open bite correction with orthognathic surgery, TADs in the anterior maxillary alveolar bone region may be used with elastomeric chains to prevent any unintended and unplanned extrusion of the maxillary incisor teeth in the preoperative orthodontics.

## Background

An anterior open bite of skeletal aetiology, sometimes referred to as a skeletal open bite, is a relatively common presentation to the joint orthognathic treatment clinic [1]. The patient’s complaints tend to be a combination of facial and smile aesthetics and potentially functional complaints, such as difficulty incising food and sometimes mastication, particularly when the open bite extends to the premolars and occasionally even the molar teeth. The presenting diagnostic features tend to include an increased lower anterior face height proportion, posterior vertical maxillary excess leading to downward and backward rotation of the mandible, and an anterior open bite [2–4]. This is usually accompanied by an incomplete lip seal in repose (sometimes referred to as lip incompetence) [5].

One of the major problems in the combined orthodontic-orthognathic surgical management of anterior open bite malocclusion is postoperative stability [1, 6–11]. There are a number of reasons why a surgically corrected anterior open bite may relapse, which include surgical instability leading to rotational relapse of the osteotomized jaws or idiopathic condylar resorption. However, one of the more common problems occurs in the preoperative orthodontic preparation. When a complete maxillary dental arch orthodontic fixed appliance is bonded and an archwire is ligated, the general tendency of modern preadjusted fixed appliances is towards unwanted and unplanned extrusion of the incisor and canine teeth as the arch is levelling. Following surgical repositioning of the jaws, the relapse potential of the extruded anterior dentition will be to intrude, which inevitably leads to some reopening of the surgically corrected anterior open bite [1].

The purpose of this case report is to demonstrate a technique using temporary anchorage devices, permitting levelling of the maxillary dental arch without extrusion of the anterior dentition, thereby improving the postoperative stability of the open bite correction.

## Case presentation

**Fig. 1:**
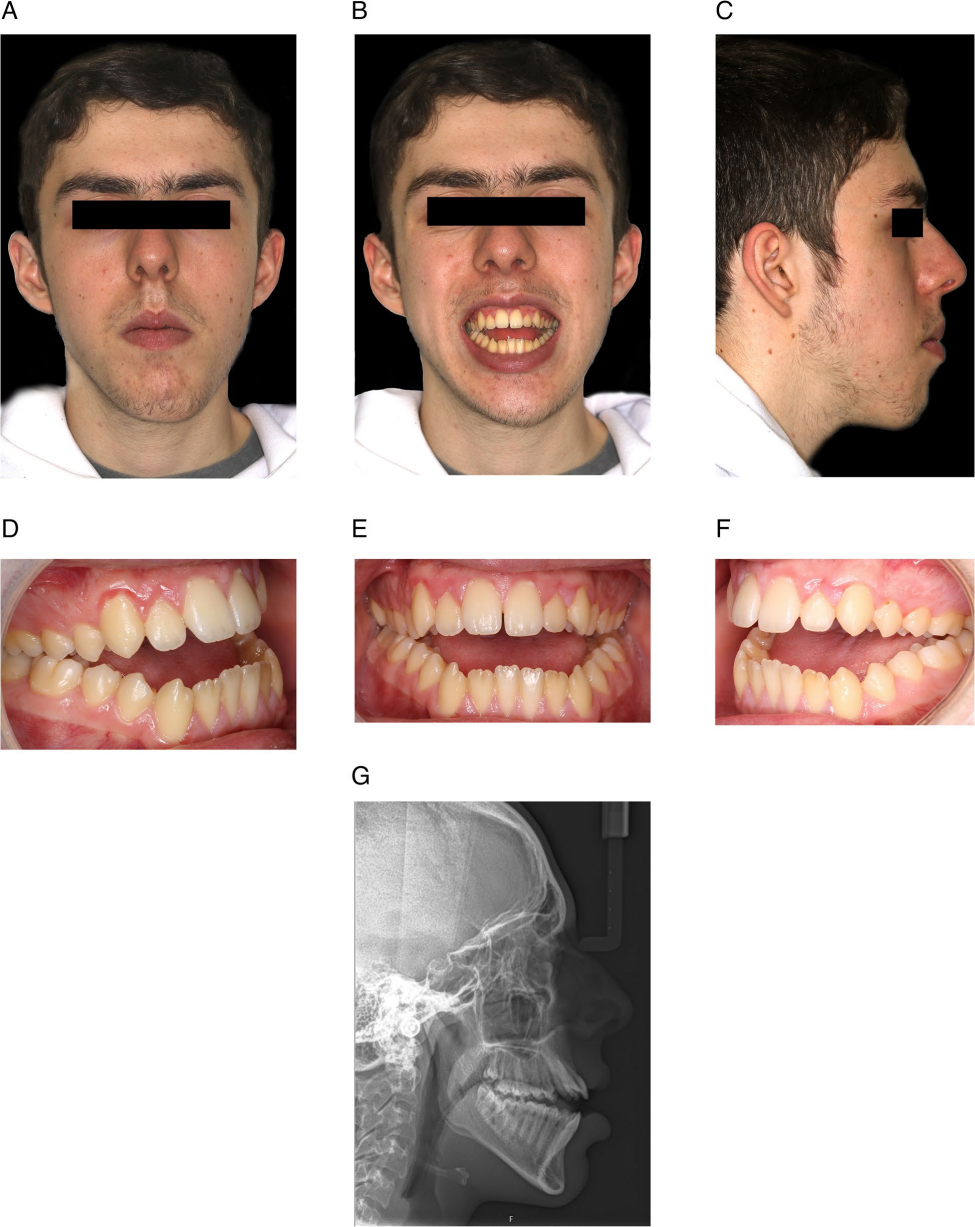
Pretreatment clinical photographs and lateral cephalometric radiograph

A 19-year-old male white Caucasian patient presented complaining of the appearance of his jaws and smile and difficulty incising food and sometimes with mastication (Fig. 1). Clinical examination demonstrated a mild Class III incisor relationship and a significant skeletal anterior open bite, measuring 6 mm, on a Class I skeletal pattern with increased lower anterior face height. Maxillary incisor exposure was 3.5 mm in repose. The mandibular unit length was increased, suggesting a potentially macrognathic mandible that had rotated clockwise during growth (i.e. a Class III rotated to a Class I). Frontal examination demonstrated mandibular asymmetry, with the patient’s chin point to his left side.

**Fig. 2:**
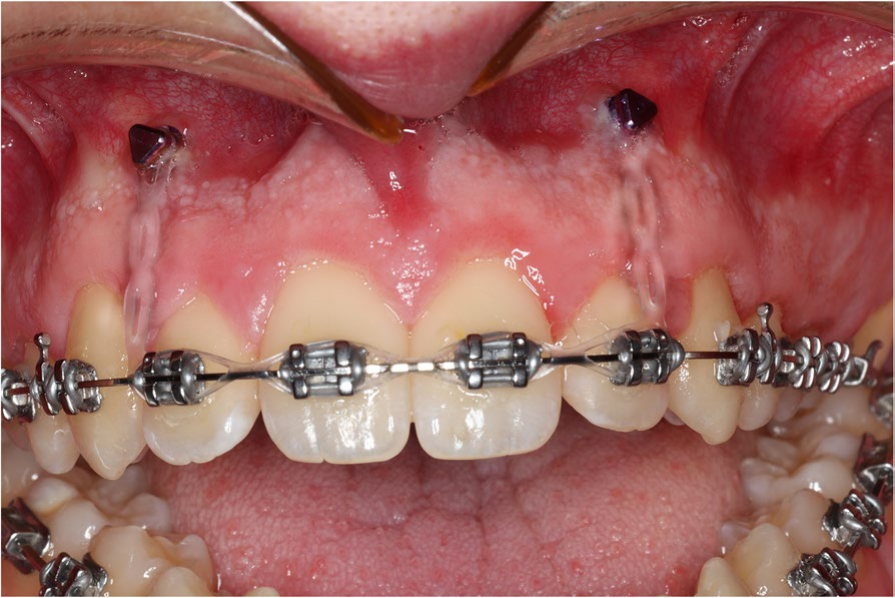
The technique involves placing TADs between the roots of the maxillary lateral incisor and canine, just below the mucogingival junction, bilaterally. Subsequently, relatively passive elastic force may be placed from the archwire, just distal to the maxillary lateral incisors, to the TADs, in order to prevent maxillary incisor extrusion during arch levelling. Alternatively, the power chain may be extended from one TAD to include the brackets on the incisor teeth and across to the opposing TAD. This latter configuration is useful if incisor proclination or palatal root torque is also planned in the preoperative orthodontic preparation

The objectives of orthodontic preparation were to align, level, decompensate, and coordinate the dental arches with fixed orthodontic appliances on a non-extraction basis. The main objective of levelling, particularly in the maxillary dental arch, was to avoid any extrusion of the anterior dentition. To achieve this aim, and counter the built-in extrusive tendency of the preadjusted appliances, two temporary anchorage devices (TADs) were placed, under local anaesthesia, in the maxillary alveolar bone in the region between the maxillary lateral incisor and canine (having assessed the interradicular space using long cone periapical radiographs), just below the mucogingival junction, bilaterally (6 mm × 1.4 mm VectorTAS miniscrews). On bonding the maxillary dental arch, light and relatively passive elastic force was placed from the archwire to the TADs in order to prevent maxillary incisor extrusion (Fig. 2). In situations where active intrusion is desired, the elastic traction may be made active by the required amount. However, in this instance, significant active intrusion was not desired, only avoidance of unplanned extrusion. Passive elastomeric chain (e.g. power chain) is preferable to stainless steel ligatures, which are an alternative but potentially more likely to cause gingival or labial trauma to the patient. Elastomeric chain was maintained throughout the alignment and levelling of the maxillary dental arch while progressing through the archwires until 0.019 × 0.025-inch stainless steel archwires were ligated. It is worth mentioning that the positioning of the TADs is important to reduce the likelihood of soft tissue irritation, screw failure, and root damage. Should the TADs fail, they can be replaced in a slightly different location, but careful planning and technique reduce this likelihood.

**Fig. 3:**
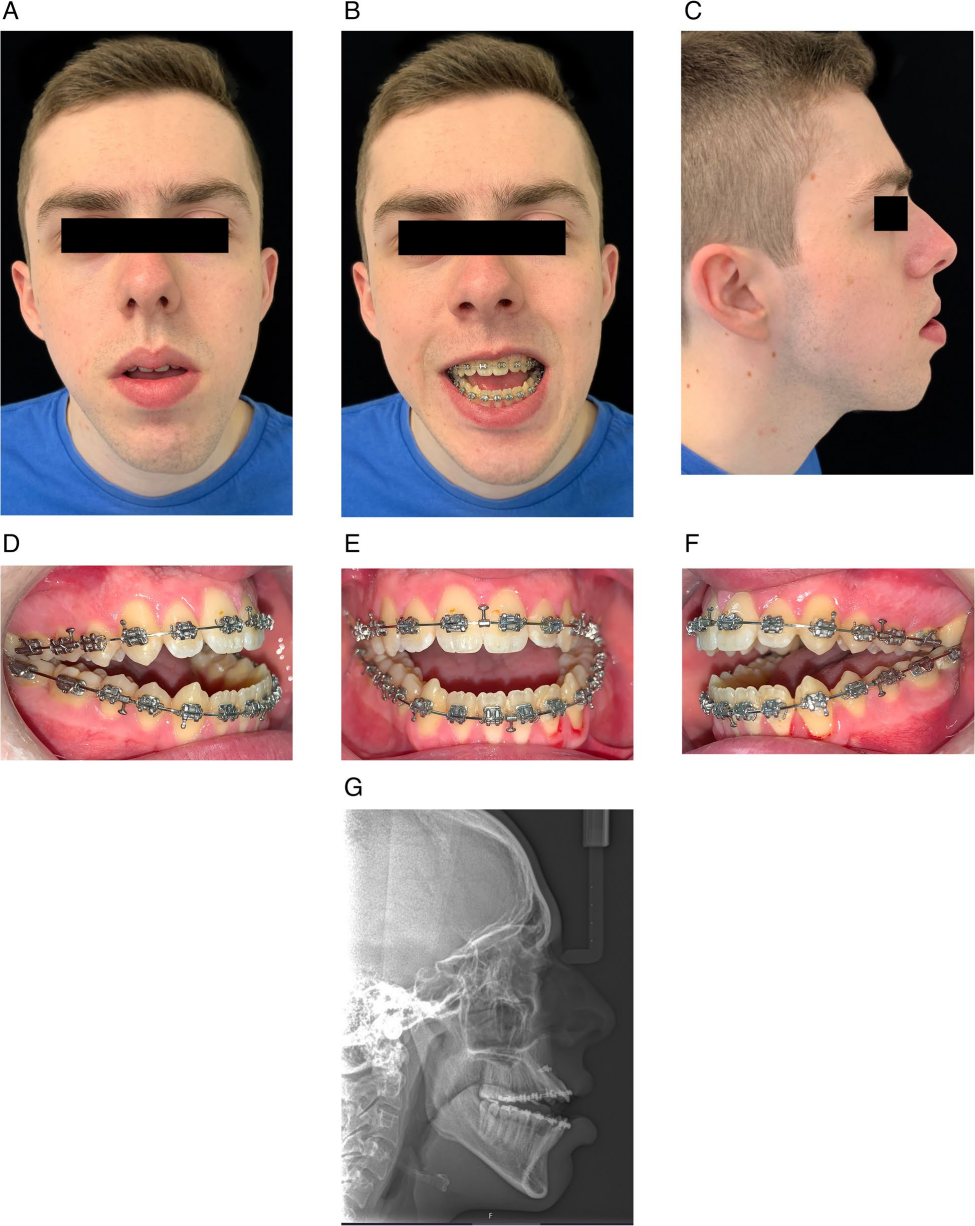
Preoperative clinical photographs and lateral cephalometric radiograph

**Fig. 4:**
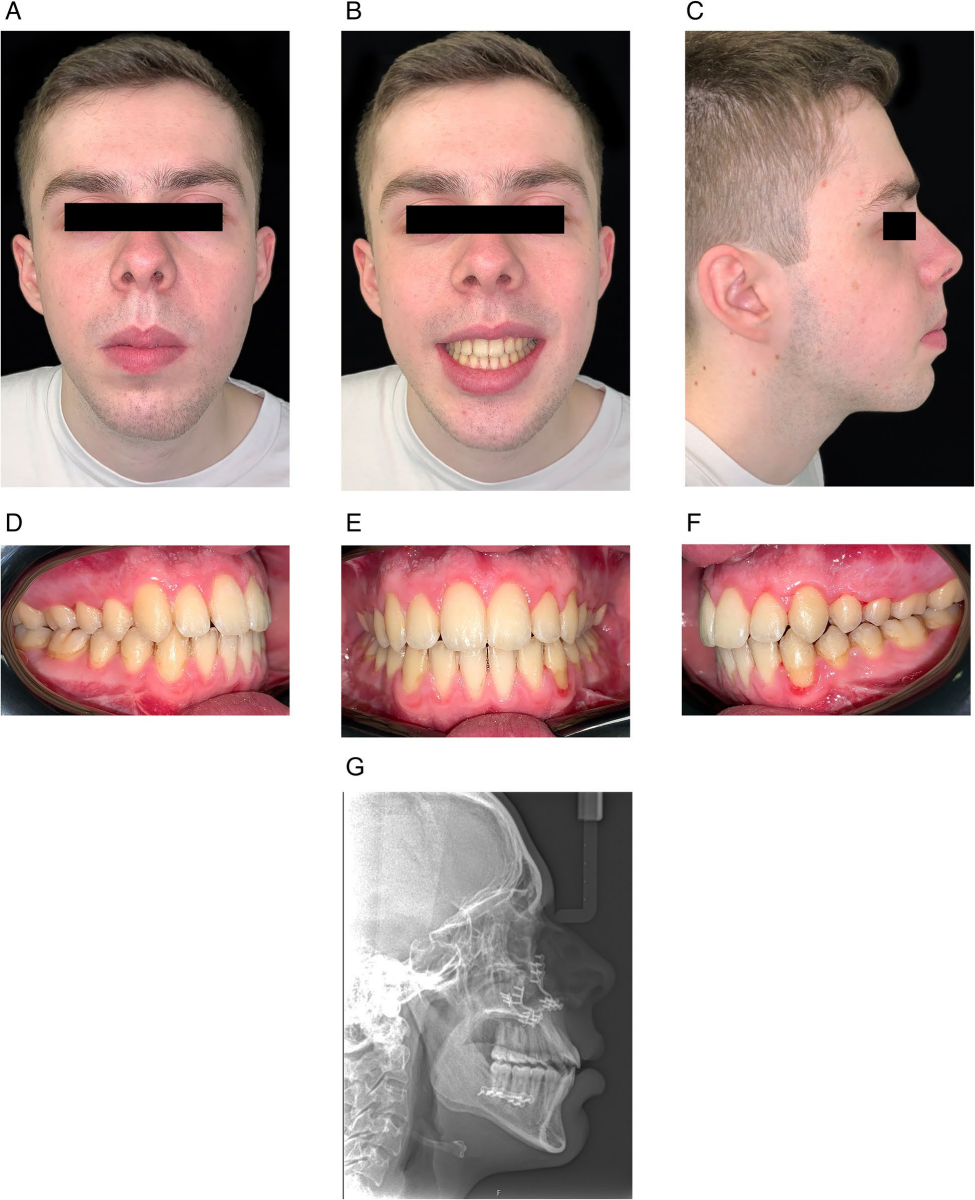
End-of-treatment clinical photographs and lateral cephalometric radiograph

When the patient was deemed ready for surgery, he was seen at a joint orthognathic planning clinic by the maxillofacial surgeon and orthodontist (Fig. 3). The clinical plan was then transferred to three-dimensional virtual surgical planning (3D-VSP), which subsequently confirmed the exact skeletal repositioning movements required. These were a Le Fort I osteotomy of the maxilla with differential posterior impaction and advancement, and mandibular forward autorotation and small asymmetrical setback of the mandibular body with bilateral sagittal split osteotomy, to achieve a Class I incisor and skeletal position. No vertical movement of the teeth was carried out or required following surgery. The TADs were maintained intraoperatively, and their position did not interfere with the Le Fort I osteotomy. We recommend maintaining the TADs postoperatively in case vertical elastics are required, although they were not required in this case. The patient was debonded 3 months after surgery, the TADs were removed, and the patient fitted with removable retainers (Fig. 4). The anterior open bite correction has remained clinically stable at 1 year following surgery.

Lateral cephalometric radiographs were all taken on the same cephalostat. The relevant comparative cephalometric values are shown in Table 1, which demonstrates an improvement in the sagittal position of the maxilla (SNA increased by 8.9°), an improvement in the maxillary incisor inclination (from the proclined 124.8 º to an average inclination of 111.4°), and a reduction in the lower anterior face height of 7.2 mm, improving the vertical facial proportions. The interlabial distance in repose reduced by 8.4 mm (from a pretreatment interlabial separation of 12.2 mm to end of treatment 3.8 mm). Further reduction in the interlabial distance would have required greater maxillary superior repositioning or increasing the height of the upper lip, neither of which were aesthetically warranted. Maxillary incisor exposure remained unchanged at 3.5 mm in repose.

**Table 1:** Comparison of cephalometric values

	Face height (nasion-menton) (mm)	LAFH (subnasale-soft tissue menton) (mm)	Interlabial distance in repose (stomion superius to stomion inferius) (mm)	Maxillary incisor inclination to true horizontal line (degrees)	Mandibular incisor inclination to mandibular plane (degrees)	SNA (degrees)	SNB (degrees)
Pretreatment	145.6	90.4	12.2	124.8	83.3	72.3	72.7
Preoperative	145.8	90.9	11.9	128.0	85.9	72.0	72.5
End of treatment	139.5	83.2	3.8	111.4	89.8	81.2	75.5

*LAFH*, lower anterior face height

Nasion (*N*), the intersection of the internasal and frontonasal sutures in the midsagittal plane

Menton, the most inferior point of the mandibular symphysis in the midsagittal plane

Subnasale, the point where the base of the nasal columella meets the upper lip in the midsagittal plane

Soft tissue menton, the most inferior point of the soft tissue chin in the midsagittal plane

Stomion superius, the most inferior point of the upper lip in the midsagittal plane

Stomion inferius, the most superior point of the lower lip in the midsagittal plane

Sella (*S*), the point representing the geometric centre of the pituitary fossa (sella turcica) in the midsagittal plane

A-point, the point at the deepest midline concavity of the maxillary alveolus between the anterior nasal spine and prosthion

B-point, the point at the deepest midline concavity of the mandibular alveolus between infradentale and pogonion

**Fig. 5:**
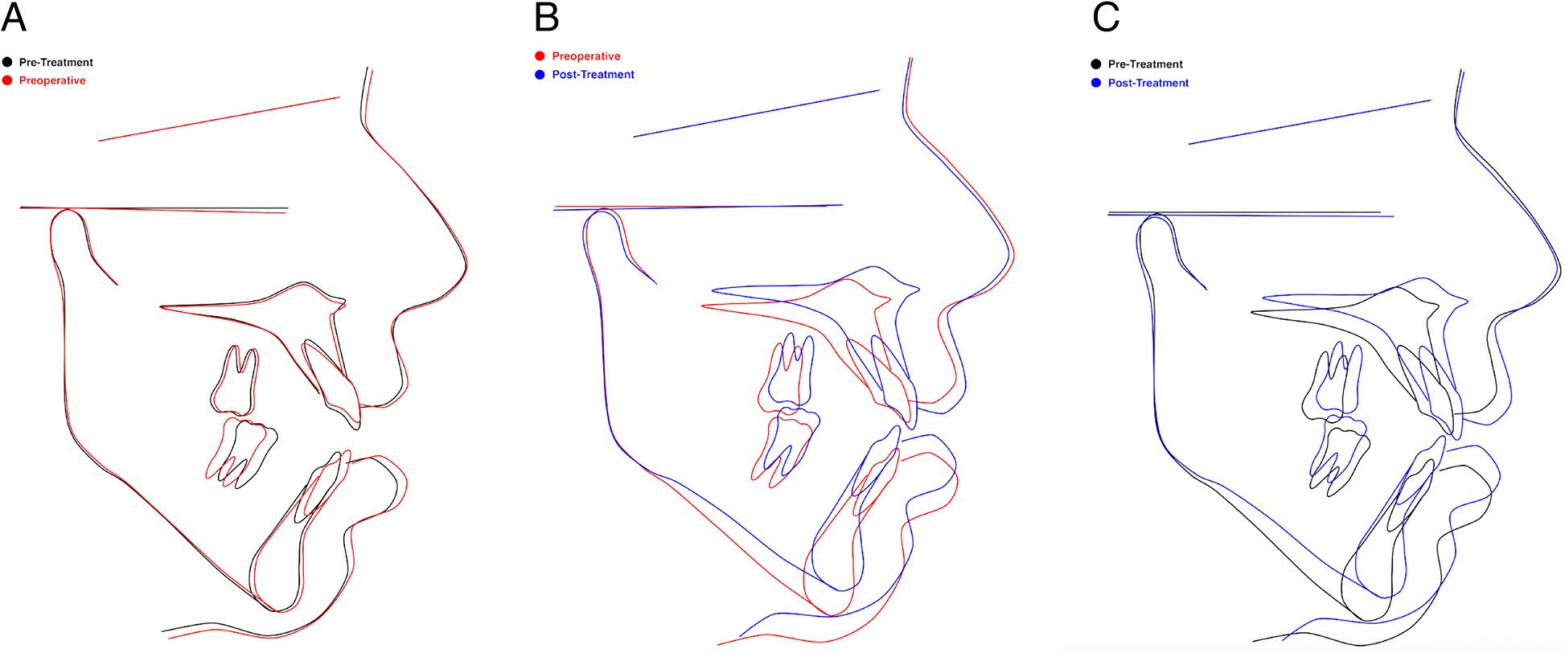
Cephalometric superimpositions. **a** Pretreatment (black) and preoperative (red) cephalometric radiographs. This superimposition demonstrates no extrusion of the anterior maxillary dentition in the preoperative arch levelling (and potentially very minor intrusion of these teeth), which is the main parameter to improve postoperative stability of the anterior open bite correction. **b** Preoperative (red) and end-of-treatment (blue) radiographs. **c** Pretreatment (black) and end-of-treatment (blue) cephalometric radiographs

Cephalometric superimpositions were subsequently undertaken using the pretreatment and preoperative cephalometric radiographs, the preoperative and end-of-treatment radiographs, and the pretreatment and end-of-treatment cephalometric radiographs (Fig. 5a, b, c). The most significant parameter for the purposes of the technical modality described in this case report is shown in Fig. 5a, which demonstrates no extrusion of the anterior maxillary dentition in the preoperative arch levelling, and potentially very minor intrusion of these teeth, which is the main parameter to improve postoperative stability of the anterior open bite correction.

## Conclusions

Stability is often regarded not as “a” problem in orthodontics but as “the” problem [12]. This is just as important, if not more so, in combined orthodontics and orthognathic surgery [6–11]. Patients carry a significant treatment burden when undergoing combined orthognathic surgery, making the stability of the final result an important determinant of quality, particularly in the long term. Extrusion of incisor teeth is one of the least stable movements in orthodontic treatment. When this is combined with skeletal repositioning in the orthognathic management of anterior open bite malocclusion, the risk of posttreatment intrusive relapse and reopening of the bite may be especially problematic. The technique described in this case report prevents unintended extrusion of the maxillary incisor teeth in the preoperative phase of orthodontic arch levelling, potentially reducing or negating the relapse potential after surgery and thereby improving the stability of anterior open bite correction with orthognathic treatment.

## Data Availability

No datasets were generated or analysed during the current study.

## Abbreviations

LAFH: Lower anterior face height
TADs: Temporary anchorage devices
3D-VSP: Three-dimensional virtual surgical planning
